# A new approach to off-gas analysis for shaken bioreactors showing high CTR and RQ accuracy

**DOI:** 10.1186/s13036-025-00480-5

**Published:** 2025-01-28

**Authors:** Andreas Schulte, Janik Brockmann, Nina Müller, Tibor Anderlei, Jochen Büchs

**Affiliations:** 1 AVT – Biochemical Engineering, RWTH Aachen University, Forckenbeckstr. 51, D-52074 Aachen, Germany; 2 Kuhner Shaker GmbH, Kaiserstr. 100, 52134 Herzogenrath, Germany; 3 Kuhner AG, Dinkelbergstr. 1, Birsfelden, CH-4127 Switzerland

**Keywords:** Respiration activity, Off-gas analysis, OTR, CTR, RQ, Shake flask, PAT

## Abstract

**Background:**

Shake flasks are essential tools in biotechnological development due to their cost efficiency and ease of use. However, a significant challenge is the miniaturization of process analytical tools to maximize information output from each cultivation. This study aimed to develop a respiration activity online measurement system via off-gas analysis, named “Transfer rate Online Measurement” (TOM), for determining the oxygen transfer rate (OTR), carbon dioxide transfer rate (CTR), and the respiration quotient (RQ) in surface-aerated bioreactors, primarily targeting shake flasks.

**Results:**

Sensors for off-gas analysis were placed in a bypass system that avoids the shaking of the electronics and sensors. An electrochemical oxygen sensor and an infrared CO_2_ sensor were used. The bypass system was combined with the established method of recurrent dynamic measurement phases, evaluating the decrease in oxygen and the increase in CO_2_ during stopped aeration. The newly developed measurement system showed high accuracy, precision and reproducibility among individual flasks, especially regarding CTR measurement. The system was compared with state-of-the-art RAMOS technology (Respiration Activity Monitoring System, see explanation below) and calibrated with a non-biological model system. The accuracy of RQ measurement was +-4% for the tested range (8% filling volume, OTR and CTR: 0–56 mmol/L/h), allowing for the determination of metabolic switches and quantitative analysis of metabolites. At ambient CO_2_ levels, a CTR resolution of less than 0.01 mmol/L/h was possible. The system was applied to the microbial model systems *S. cerevisiae*, *G. oxydans*, and *E. coli*. Physiological states, such as growth vs. protein production, could be revealed, and quantitative analysis of metabolites was performed, putting focus on RQ measurements.

**Conclusions:**

The developed TOM system showcases a novel approach to measuring OTR, CTR, and RQ in shaken bioreactors. It offers a robust and accurate solution for respiration activity analysis. Due to its flexible design and tunable accuracy, it enables measurement in various applications and different shake flasks.

**Supplementary Information:**

The online version contains supplementary material available at 10.1186/s13036-025-00480-5.

## Background

Shaken bioreactors, such as microplates and shake flasks, are widely used in screening, media- and process development or for pre-cultures in production processes. Their frequent use can be attributed to their easy use and the ability to run them in parallel at low costs [1]. Compared to large scale fermenters, process analytical tools for shake flasks must be small and measure several flasks in parallel. This comes with the drawback of limited monitoring possibilities. However, online measurement tools for shaken bioreactors have emerged during the last two decades, while these tools have been used in stirred tank reactors for a long time. Measurement of optical density, pH, fluorescence, dissolved oxygen tension (DOT) and off-gas analysis have been successfully applied to microplate- and shake flask cultivations and have been commercialized in some cases [2–4]. Klöckner and Büchs [1] have reviewed most of the technologies. Process analytical technology (PAT) in small scale enhances the quality by design (QbD) paradigm for good process understanding, robust operation and risk-reduced process development [5–7].

Respiration activity measurement via off-gas analysis has a special importance within those process analytical tools, as it is non-invasive to the liquid broth, and it provides absolute numbers with clear units. The values can e.g. be used for material balances. Common off-gas analysis focuses on measurement of oxygen and carbon dioxide measurement and yields the oxygen transfer rate (OTR), carbon dioxide transfer rate (CTR) and the respiration quotient (RQ = CTR/OTR). OTR and CTR are quantitative measures of the physiological state of a culture, mostly presented in the unit $\:\left[\frac{mmol\:gas}{volume\:culture\:broth\:\times\:\:time\:}\right]$. This normalization to the liquid volume makes measurements of the respiration activity comparable throughout all scales and between different reactors. Any volatile compound being transferred from the headspace to the liquid broth or vice versa could be monitored, such as ethylene or ethanol [8, 9]. In anaerobic digestion, the biochemical methane potential (BMP) of substrates is of great interest and is usually conducted according to VDI 4630. Also, hydrogen and carbon monoxide measurement in small scale was reported recently [10–12].

Different techniques for O_2_ and CO_2_ measurement in bioreactors have been reviewed in the past [13, 14]. A common off-gas analysis method for bioprocesses is the analysis of gas partial pressure in the headspace or the off-gas of a bioreactor. If the flow rate (F_in_) and gas concentrations (*p*_*CO2,in*_, *p*_*O2,in*_) at the reactor inlet are known and the output gas stream (F_*out*_) and headspace gas partial pressure (*p*_*CO2*_, *p*_*O2*_) are measured, a simple molar balance can be drawn to yield OTR and CTR, as specified in Eqs. 1 and 2.

1 $$\:\frac{d{p}_{O2}}{dt}\times\:{V}_{g}=-OTR\:\times\:{V}_{l}\times\:R\times\:T+{F}_{in}\times\:{p}_{O2,in}-{F}_{out}\times\:{p}_{O2}$$

2 $$\:\frac{d{p}_{CO2}}{dt}\times\:{V}_{g}=CTR\:\times\:{V}_{l}\times\:R\times\:T+{F}_{in}\times\:{p}_{CO2,in}-{F}_{out}\times\:{p}_{CO2}$$

*V*_*g*_ being the reactor head space gas volume and *V*_*l*_ the liquid volume. This technology is dependent on the accurate determination of concentrations and volume flows, as seen from Eqs. 1 and 2. This approach is usually applied to stirred tank reactors with sophisticated analytics and has also been applied on shake flask scale for a commercial product (BlueSens gas sensor GmbH). Takahashi et al. [15] detected O_2_ and CO_2_ concentrations in shake flasks with a circular direct monitoring and sampling system (CDMSS). Ge et al. [16]. have presented a method to detect O_2_ and CO_2_ concentrations in the off-gas of actively aerated shake flask and in the headspace of shake flasks, ventilated with a milk filter. However, both groups did not convert detected concentrations to OTR and CTR, respectively. Concentrations in the headspace are depending on the flask ventilation as well as biomass amount and are, thus, not directly comparable, when cultivation conditions, sterile closures or aeration setups are changed.

A second possibility for on-line measurement of OTR and CTR is a recurring cycle of aeration and stopped aeration of the shake flask headspace [17]. In this case, OTR and CTR can be calculated from the dynamic decrease of the oxygen partial pressure and the increase of the carbon dioxide partial pressure over time during stopped aeration (Eqs. 3 and 4). The RAMOS (Respiration Activity Monitoring System) is working according to this method [17]. The oxygen and carbon dioxide balance then simplifies to:

3 $$\:\frac{d{p}_{O2}}{dt}\times\:{V}_{g}=-OTR\:\times\:{V}_{l}\times\:R\times\:T$$

4 $$\:\frac{d{p}_{CO2}}{dt}\times\:{V}_{g}=CTR\:\times\:{V}_{l}\times\:R\times\:T$$

The advantage of Eqs. 3 and 4, compared to Eqs. 1 and 2, respectively, is that the terms containing incoming and outcoming gas streams are erased. Measurement of both quantities is not necessary. Only the change in oxygen partial pressure $\:\frac{d{p}_{O2}}{dt}$ and carbon dioxide partial pressure $\:\frac{d{p}_{CO2}}{dt}$ is decisive for the determination of OTR and CTR. The longer the time of stopped aeration is (measurement time, t_meas_) the more precise the measurement of OTR and CTR will be. Thus, this method can even be applied at low respiration activity or for detection of trace gases. This was successfully shown for oxygen and ethylene traces in shake flask plant cell suspension cultivations [9] and for carbon dioxide and hydrogen in anaerobic shake flask cultivations [10, 12]. Also, low respiration mammalian cell cultures have been monitored successfully [18–20]. Hansen et al. [21] suggested a combination of Eq. 1 to 4 to increase the data density for the RAMOS.

The OTR can also be calculated from DOT measurements (Eq. 5) but requires the knowledge of the volumetric mass transfer coefficient (k_L_a), the oxygen solubility in the culture medium (L_O2_) and the partial pressure of oxygen in the headspace (p_O2_). The k_L_a can be obtained from literature, as reviewed by Klöckner and Büchs [1]. L_O2_ can be assumed from literature [22, 23] but can be subject to variation throughout a cultivation [24]. The oxygen partial pressure in the headspace depends on aeration or ventilation through the sterile barrier [25].

5 $$\:OTR={k}_{L}a\times\:{L}_{O2}({p}_{O2}-\frac{DOT}{100}{p}_{O2,cal})$$

$\:{p}_{O2}$ is the oxygen partial pressure in the headspace, $\:{p}_{O2,\text{c}\text{a}\text{l}}$ is the partial pressure in the headspace during calibration of the DOT sensor (usually 0.21 bar). Care must be taken, not to alter the flask hydrodynamics, when measuring the DOT inside the liquid broth (e.g. with relatively large Clark-electrodes), as this may result in erroneous measurement results [26].

OTR and CTR analysis in shake flasks have been applied for various tasks. For example, knowledge of oxygen supply is essential for scale-up and scale-down [27–31]. Diederichs et al. [32] and Sparviero et al. [33] have revealed quality variations of complex medium ingredients (yeast extract) via respiration measurements in shake flasks. RQ measurement may also reveal metabolic switches [34]. In cell culture applications, knowledge of the oxygen uptake can help to optimize feeding strategies [35–37] and gives a direct measure for the cellular activity [36, 38]. Furthermore, viable cell density, glucose consumption and even lactate formation can be estimated from off-gas analysis [19]. Off-gas analysis has also been applied for the characterization of shaken bioreactors [39] and numerous examples for media development [40–43] have been published for batch and fed-batch applications.

The goal of this study is the development of an off-gas online measurement tool for determination of OTR, CTR and RQ in surface-aerated bioreactors with shake flasks as primary application example. The following requirements for this novel measurement tool were defined: (1) parallelization for simultaneous monitoring of up to 16 individual bioreactors, (2) low cost, (3) robust to intense shaking environment, (4) small footprint on the shaker tray and (5) high precision and accuracy even at small working volumes or low cell counts. To achieve these requirements, recurring phases of aeration and stopped aeration are realized, in literature often referred to as RAMOS. Gas sensors were placed in a bypass and the measuring gases were supplied by microfluidic gas pumps. The O_2_- and CO_2_ sensors, the gas pumps and the electronics are mounted at the ceiling of the shaker cabinet, resulting in maximum space for shake flasks on the shaker tray and low wear of the sensors, thus robust operation. Shake flasks were actively aerated at defined flowrates. The working principle of TOM (Transfer rate Online Measurement) is described, and its performance is validated by exemplary microbial cultivations presented before. It is not the aim of this work to generate new findings on microbial physiological behavior.

## Results

**Fig. 1:**
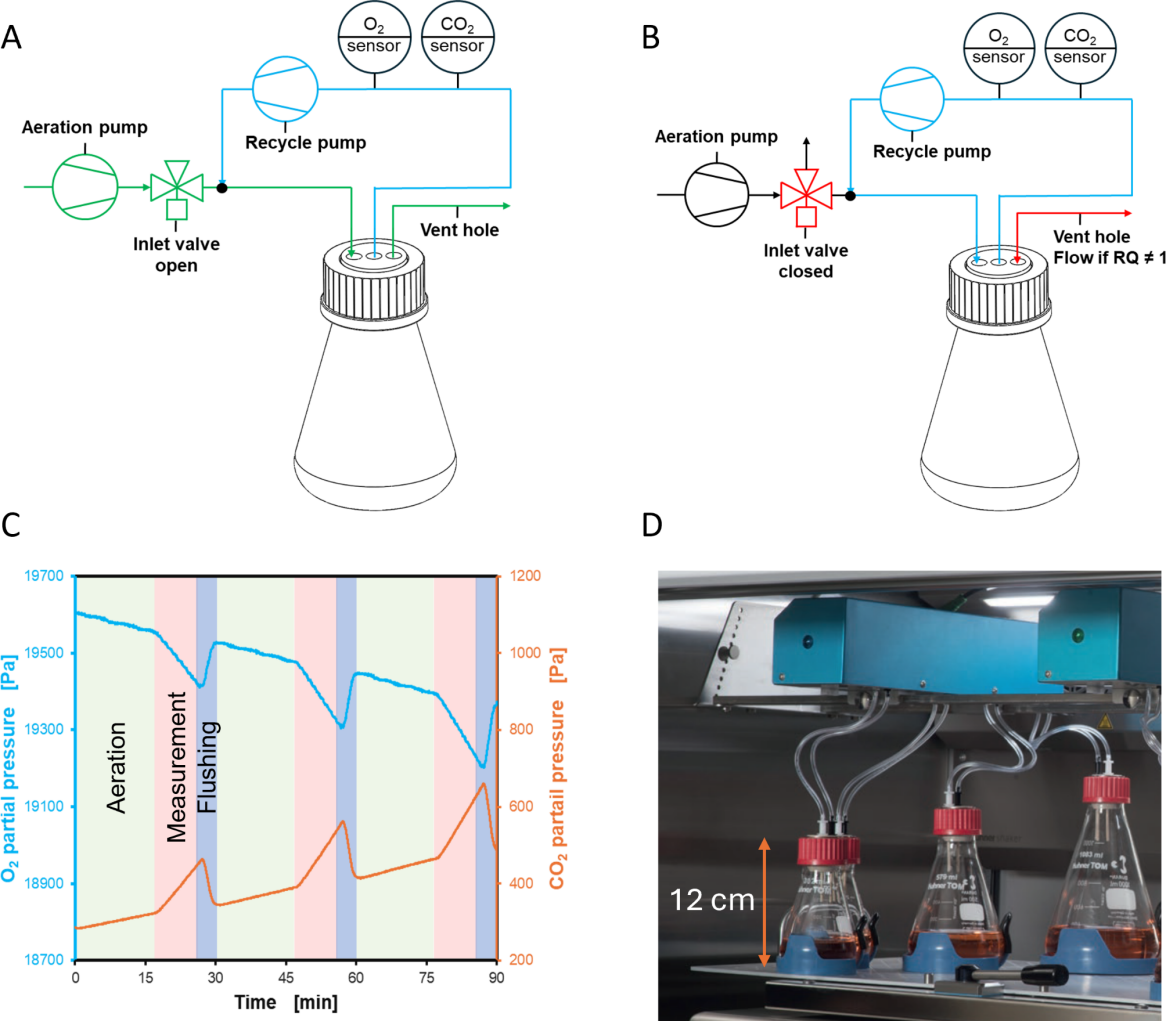
Illustration of the flow scheme and measurement cycle. The TOM device measures the O_2_ and CO_2_ partial pressure of the shake flask’s headspace and calculates the OTR and CTR, respectively. Headspace gas is continuously withdrawn with a microfluidic pump (Recycle pump, approx. 15 mL/min), analyzed by an O_2_ and CO_2_ sensor, and recycled to the shake flask (blue lines). **A** Shake flasks are aerated with a self-priming pump in the aeration and flushing phase (Aeration pump, adjustable aeration rate). The gas outlet is designed as an open vent hole. **B** In the measurement phase, aeration is stopped by switching an 3/2-way inlet valve. CO_2_ partial pressure and O_2_ partial pressure change during the measurement phase according to cell respiration. In general, CO_2_ increases and oxygen decreases during the measurement phase (see Fig. 1C). OTR and CTR is calculated based on these partial pressure changes over time (see Eqs. 3 and 4). The vent hole is always open to allow for gas exchange in case of more CO_2_ is produced than O_2_ consumed (RQ > 1) or less CO_2_ is produced than O_2_ consumed (RQ < 1) **C** Illustration of the gas concentration changes during the measurement cycle. Aeration phase (light green, valve open, aeration), measurement phase (light red, valve closed, no aeration) and flushing phase (dark blue, valve open, aeration at higher flow) are recurrently repeated. **D** Picture of TOM with different sized glass flasks

The newly developed TOM measurement system is based on the RAMOS. Major changes have been introduced during this study to improve performance, handling and robustness. In the following, these changes from RAMOS to TOM are presented and evaluated using an intermediate system. Additional file 1 illustrates the development from RAMOS (Additional file 2 A) to TOM (Fig. 1) and states which system was used to obtain the according experimental data.

### Sensor technology for CTR measurement

**Fig. 2:**
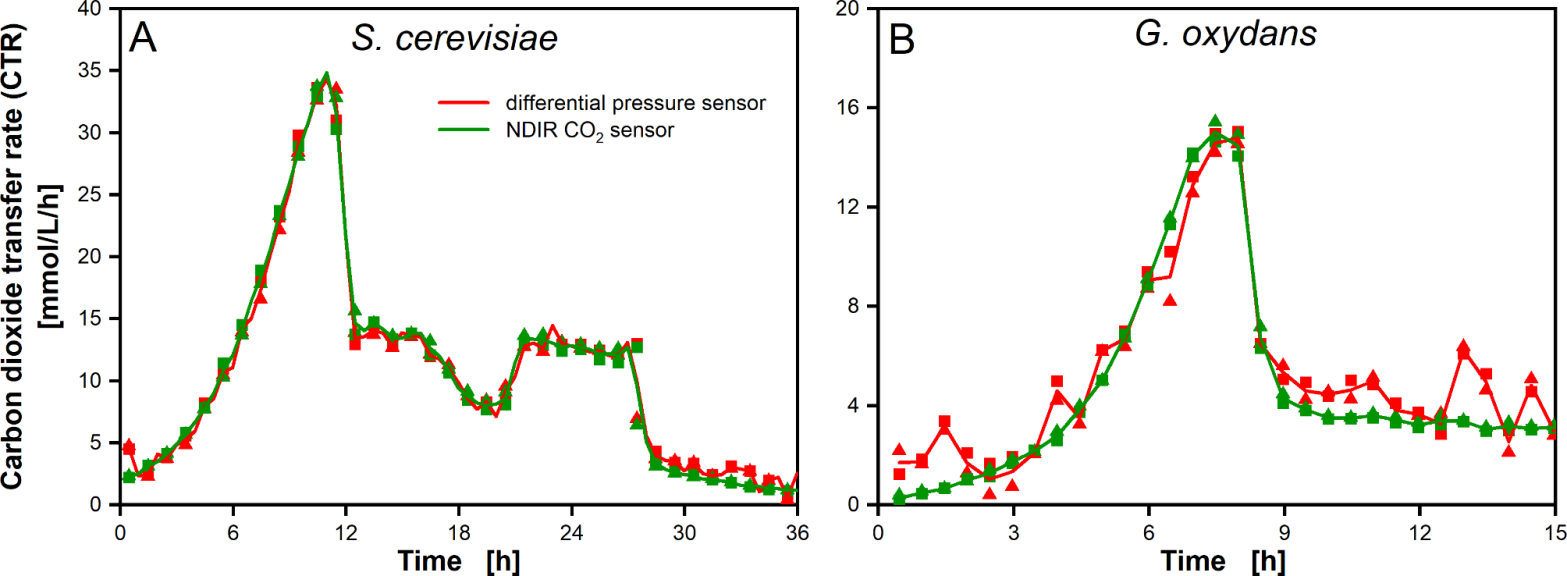
Comparison of differential pressure sensor (∆p sensor) and non-dispersive infrared CO_2_ sensor (NDIR CO_2_ sensor) for CTR measurement. CTR over time determined with ∆p sensor (red) (as described by Anderlei et al. [17] and NDIR CO_2_ sensor (green). For comparison of both methods, a ∆p sensor was included in the measurement setup as illustrated in Additional file 2 B. The open vent hole was replaced by a switchable outlet valve, to allow for gas tightness. Data points are presented as duplicate measurements in two individual shake flasks (square and triangle). The solid line connects the average of these two data points. **A*** Saccharomyces cerevisiae* (*S. cerevisiae*) cultivation in yeast extract peptone medium (YEP-medium) containing 20 g/L glucose. Operation conditions: shaking frequency *n* = 200 rpm, shaking diameter d_0_ = 50 mm, liquid filling volume V_L_ = 25 mL, 250 mL shake flask, temperature T = 30 °C, measurement time t_meas_ = 6 min; **B*** Gluconobacter oxydans* (*G. oxydans*) in Jülich-medium containing 80 g/L mannitol. Operation conditions: *n* = 350 rpm, d_0_ = 50 mm, V_L_ = 10 mL, 250 mL shake flask, T = 30 °C, t_meas_ = 6 min

As mentioned above, the TOM measurement principle is based on the intermittent method presented before for the RAMOS [17]. The RAMOS measurement system is equipped with an inlet valve at the flask inlet and an outlet valve at the flask outlet (see Additional file 2 A). When both valves are closed in the measurement phase, the shake flask constitutes a closed volume. For this defined volume, molar balances for O_2_ and CO_2_ can be made (see Eq. 3 and Eq. 4). A differential pressure sensor (∆p sensor) is used as a surrogate sensor for the measurement of the CO_2_ partial pressure, assuming that only O_2_ and CO_2_ are transferred between liquid and gas phase in the shake flask. However, an NDIR sensor has higher resolution and is specific to CO_2_. A change in temperature, humidity, other volatile compounds or leakage may affect the CTR measurement with a ∆p sensor, in contrast to an NDIR CO_2_ sensor. To address these drawbacks, the ∆p sensor was compared to an NDIR CO_2_ sensor in a modified TOM (see Additional file 2 B). This modified TOM comprises both NDIR CO_2_ and ∆p sensors for CTR measurement (and oxygen sensor for OTR measurement) and a valve at the flask outlet, to achieve a gas-tight setup. Both sensors were attached to the same shake flask. Figure 2A shows the CTR over time for a *S. cerevisiae* cultivation on glucose as primary carbon source. This serves as an example for a process with high carbon dioxide formation (max. 35 mmol/L/h at 25 mL filling volume = 0.875 mmol/h at approx. 10 h). No distinct difference between ∆p sensor and NDIR CO_2_ sensor based CTR can be observed at CTR levels above 10 mmol/L/h at 25 mL filling volume (0.25 mmol/h). At lower respiration (from 28 h on), measurements from both sensors differed slightly. Figure 2B shows the CTR over time for a *G. oxydans* cultivation. This serves as an example of a low carbon dioxide forming microbial system (max. 16 mmol/L/h at 10 mL filling volume = 0.16 mmol/h). Here, the ∆p sensor based CTR shows fluctuations, while corresponding NDIR CO_2_ sensor based CTR shows a rather steady course. Fluctuations in ∆p sensor based CTR were attributed to ambient influences (e.g., temperature fluctuations or ambient pressure changes) rather than sensor or flask specific issues, as they occur for both duplicate data points (measurements in two individual shake flasks). NDIR CO_2_ sensors detect light absorption by CO_2_ molecules, according to Lambert Beer’s theorem [44]. Thus, these sensors are most sensitive at low CO_2_ partial pressure, explaining the superior resolution at low respiration activity.

### O_2_ and CO_2_ balancing

The final TOM design, as shown in Fig. 1, encloses a vent hole to release off-gas, when the shake flask is aerated. This vent hole is also open to the environment during the measurement phase. Thus, the shake flask cannot be seen as a closed balance space. If the cells’ CO_2_ production is higher than their O_2_ consumption (RQ > 1), a convective gas flow escapes from the vent hole. This would yield an overestimated OTR and an under estimated CTR, according to Eqs. 3 and 4, but still present RQ > 1. If CO_2_ production is lower than O_2_ consumption (RQ < 1), air is advected from the ambient environment. This would yield an underestimated OTR and an overestimated CTR, according to Eqs. 3 and 4, but still present RQ < 1. If RQ = 1, Eqs. 3 and 4 would yield correct OTR and CTR. Consequently, the RQ based on Eqs. 3 and 4 is decisive for air from the ambient environment being advected or off-gas from the shake flask flowing out to the ambient environment. To account for this, Eqs. 3 and 4 are extended for the convective flow through the vent hole$\:\:{{F}_{out}}^{\text{*}}$. $\:{{p}_{O2}}^{\text{*}}$ and $\:{{\:p}_{CO2}}^{\text{*}}$ represent the partial pressure in the shake flask or the ambient environment and are case dependent.

6 $$\:{{F}_{out}}^{*}=\left(CTR-OTR\right)\times\:{V}_{l}\times\:\left(\frac{R\times\:T}{{p}_{ges}}\right)$$

7 $$\:-{{F}_{out}}^{*}{{p}_{O2}}^{*}-OTR\times\:{V}_{l}\times\:R\times\:T=\frac{d{p}_{O2}}{dt}\times\:{V}_{g}\:\:$$

8 $$\:-{{F}_{out}}^{*}{{p}_{CO2}}^{*}+CTR\times\:{V}_{l}\times\:R\times\:T=\frac{d{p}_{CO2}}{dt}\times\:{V}_{g}\:$$

**Fig. 3:**
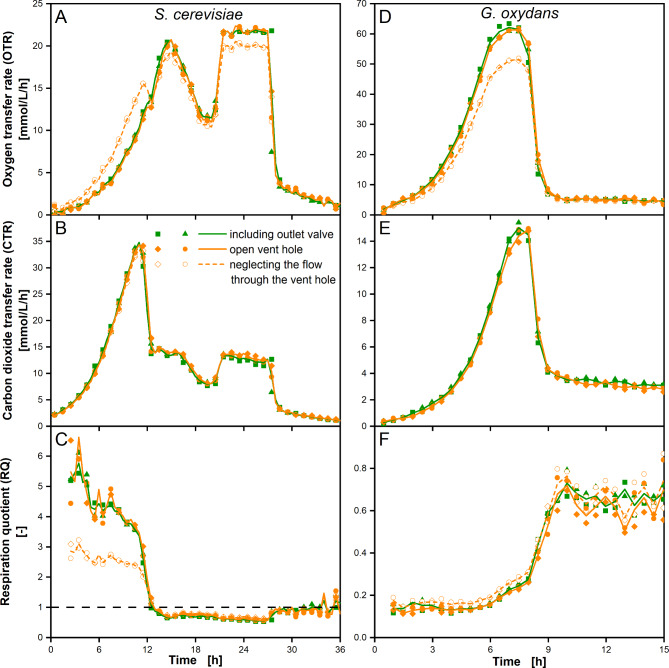
Comparison of respiration measurement using an open vent hole or an outlet valve. OTR, CTR and RQ was determined for *S. cerevisiae* and *G. oxydans* cultivations in a TOM setup with open vent hole (as in Fig. 1) and with outlet valve (as shown in Additional file 2 B) for validation of the former. Results including outlet valve are presented as green lines and closed green triangles and squares. Results with open vent hole are presented as solid orange lines and solid orange circles and diamonds. Neglection of the convective flow through the open vent hole in the CO_2_ and O_2_ balance leads to results shown as dashed orange line and open orange circles and diamonds. NDIR CO_2_ sensors were used for CTR and RQ measurement in both setups. Data points are presented as duplicate measurements in two individual shake flasks. The lines connect the average of these two data points. **A**, **B**, **C*** S. cerevisiae* cultivation in YEP-medium containing 20 g/L glucose. Operation conditions: *n* = 200 rpm, d_0_ = 50 mm, V_L_ = 25 mL, 250 mL shake flask, T = 30 °C; in **C** RQ = 1 is indicated as horizontal dashed line. **D**, **E**, **F*** G. oxydans* in Jülich-medium containing 80 g/L mannitol. Operation conditions: *n* = 350 rpm, d_0_ = 50 mm, V_L_ = 10 mL, 250 mL shake flask, T = 30 °C, t_meas_ = 6 min

As presented in Fig. 3, setups with outlet valve (green solid line) and open vent hole (orange solid line) were compared, but did not reveal differences in OTR and CTR measurement performance. However, neglecting the convective flow through the vent hole leads to major differences in OTR measurements (orange dashed line). In this case, the OTR is overestimated at RQ > 1 (first 12 h in *S. cerevisiae* cultivation) and underestimated at RQ < 1 (from 12 h on in *S. cerevisiae* cultivation and throughout *G. oxydans* cultivation). However, the convective flow through the vent hole has only minor impact on CTR calculation in the presented data (Fig. 3B and E). The CO_2_ concentration in the ambient environment and the shake flask is much lower than the O_2_ concentration. Hence, the convective molar flow of CO_2_ is lower than that of O_2_, resulting in a lower relevance and finally in a lower measurement deviation. According to the presented data and with compensation of the convective flow through the vent hole, the shake flask outlet can be designed as open vent hole, if a designated CO_2_ sensor is used for CTR measurement. This option was already indicated in Patent EP 0 905 229 B1 [45].

### Performance evaluation

Accuracy and repeatability of the TOM system was evaluated according to an adapted calibration setup, suggested by Schulte et al. [9]. A constant and defined flow of O_2_-depleted and CO_2_-enriched gas (4% CO_2_/17% O_2_ or 16% CO_2_/5% O_2_) is fed to a shake flask via a separate port. This mimics respiration at a known transfer rate. The fed gas concentrations are chosen such that OTR and CTR (named OTR_set_ and CTR_set_ in the following) are supposed to be equal. Consequently, the expected RQ value is 1.

**Fig. 4:**
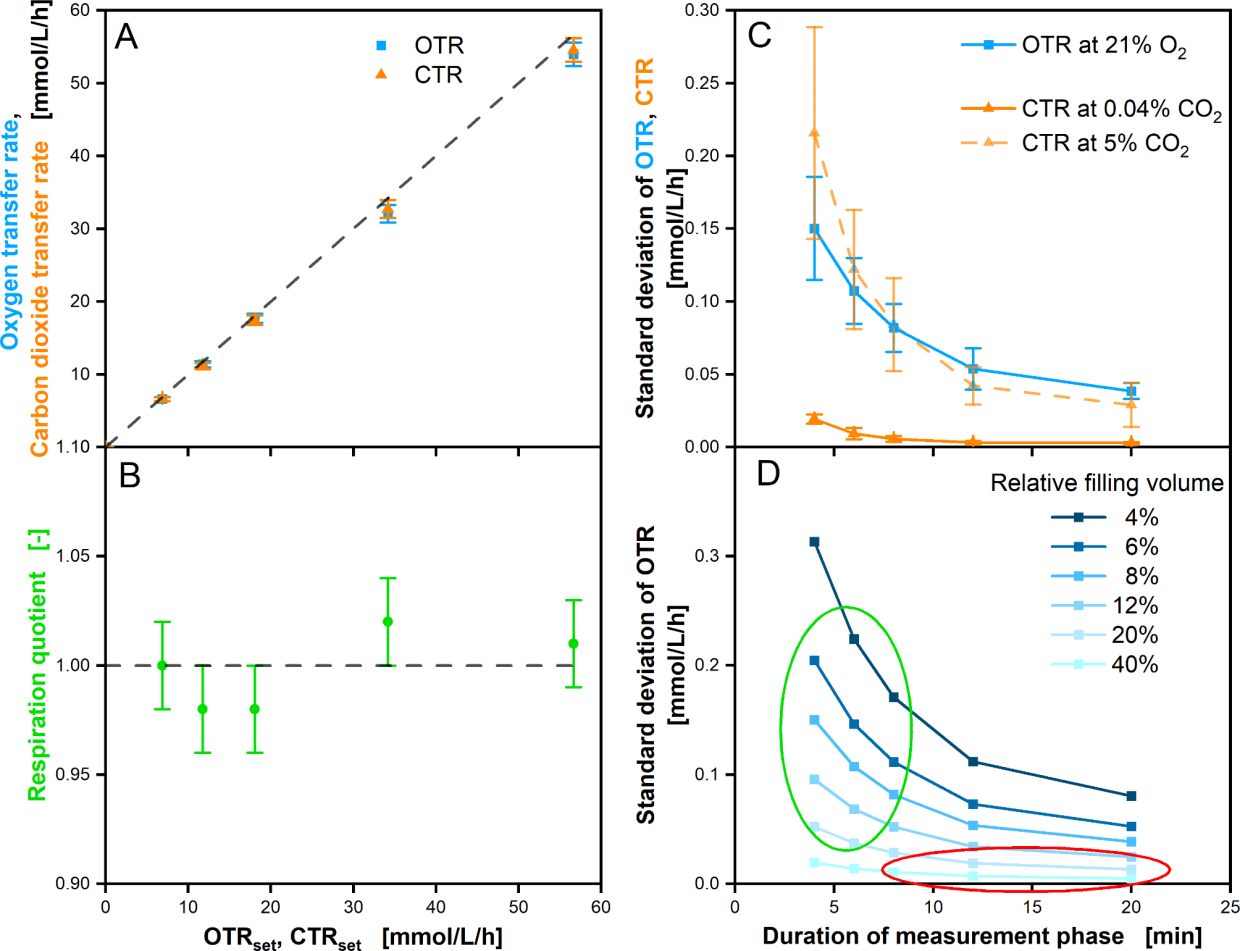
Performance of TOM in respiration measurement. Performance measurements were conducted as described by Schulte et al. [9]. Defined respiration activity was simulated by feeding a defined rate of CO_2_-enriched and O_2_-depleted gas to individual shake flasks, in the following referred to as OTR_set_ and CTR_set_, respectively. **A** OTR and CTR determined with TOM at different OTR_set_ and CTR_set_. Operation conditions: *n* = 180 rpm, d_0_ = 50 mm, V_L_ = 10 mL water, 250 mL shake flask, T = 30 °C, t_meas_ (10 min duration of measurement phase) **B** RQ accuracy and reproducibility derived from OTR and CTR data in **A**. Data points in **A** and **B** are an average of 32 measurements from 8 individual shake flasks (4 measurements each flask). Standard deviations between individual flasks are presented as error bars. **C** Standard deviation of OTR and CTR determined with TOM at 21% O_2_, 0% CO_2_ and 5% CO_2_ at varying duration of measurement phase. Operation conditions: *n* = 180 rpm, d_0_ = 50 mm, V_L_ = 20 mL water, 250 mL shake flask, T = 30 °C. **D** Standard deviation of OTR at varying relative filling volumes from 4–40% over duration of the measurement phase. The green oval indicates common operating conditions for microbial cultivations, the red oval indicates common operating conditions for mammalian and plant cell cultivations

Figure 4A shows measured OTR and CTR values over OTR_set_ and CTR_set_ (6 min measurement time). Throughout the tested measurement range from 0 mmol/L/h to 57 mmol/L/h, the averaged (4 measurements) OTR and CTR were always measured in a range of + 0…-5% of the set values. This small systematic negative deviation from the set values is within the expected deviation caused by the experimental setup. Sources for deviation may be the accuracies of the mass flow controller or a slightly underestimated shake flask gas volume. Therefore, the presented data may serve as a benchmark for the overall accuracy of the TOM system.

Deviations caused by the experimental setup influence OTR and CTR in the same way, as both sensors are attached to the same flask. Consequently, the RQ can serve as a measure for determination of the measuring accuracy. As seen from Fig. 4B, within the tested measurement range, the averaged measured RQ was within +-4% of the set value (accuracy). The RQ value measurement varied between individual shake flasks at a standard deviation of +-2%, presented as error bars (reproducibility between flasks).

Figure 4C shows the average standard deviation in OTR and CTR measurement related to the duration of the measurement phase (t_meas_). This serves as a measure for the precision in subsequent measurements and is an indicator of the resolution of the TOM system. Error bars indicate the reproducibility between individual shake flasks. The standard deviation decreases with the duration of the measurement phase. Therefore, the duration of the measurement phase is the main parameter to adjust the desired measurement resolution. The CTR resolution generally decreases at higher CO_2_ concentration. The standard deviation in CTR measurement is approx. 10-fold higher at 5% CO_2_ than at 0.04% CO_2_. This can be attributed to the decreasing resolution of NDIR CO_2_ sensors at high CO_2_ concentration. To our knowledge, a standard deviation in CTR measurement < 0.02 mmol/L/h (solid orange line) in low respiration CTR measurement has not been reported so far for small scale shaken bioreactors. It is a result of sensitive NDIR CO_2_ partial pressure measurement and CTR measurement at stopped aeration (RAMOS). Data in Fig. 4C were measured at a relative filling volume of 8% (20 mL) in a shake flask with 250 mL nominal volume. Resolution scales inversely proportional to filling volume. Consequently, the resolution could be lower or higher depending on the filling volume.

Figure 4D exemplifies the correlation between standard deviation and duration of measuring phase, while the “8%” graph (20 mL filling volume) is the original measured data, and all other graphs are computed. The red circle marks the common range for mammalian or plant cell culture cultivations (low respiration = long measurement phase and high filling volume) [9, 20, 46], while the green circle marks the common range for microbial cultivations (high respiration = short measurement phase and low filling volume.

### Exemplary cultivations

Two model cultivations have been chosen to exemplify the abilities of the newly developed measurement system, focusing mainly on the potential of CTR and RQ measurement. Variations of previously published cultivations are analyzed in the context of online off-gas analysis. The investigation of physiological phenomena was not part of this study.

### *G. oxydans* growth on mannitol

**Fig. 5:**
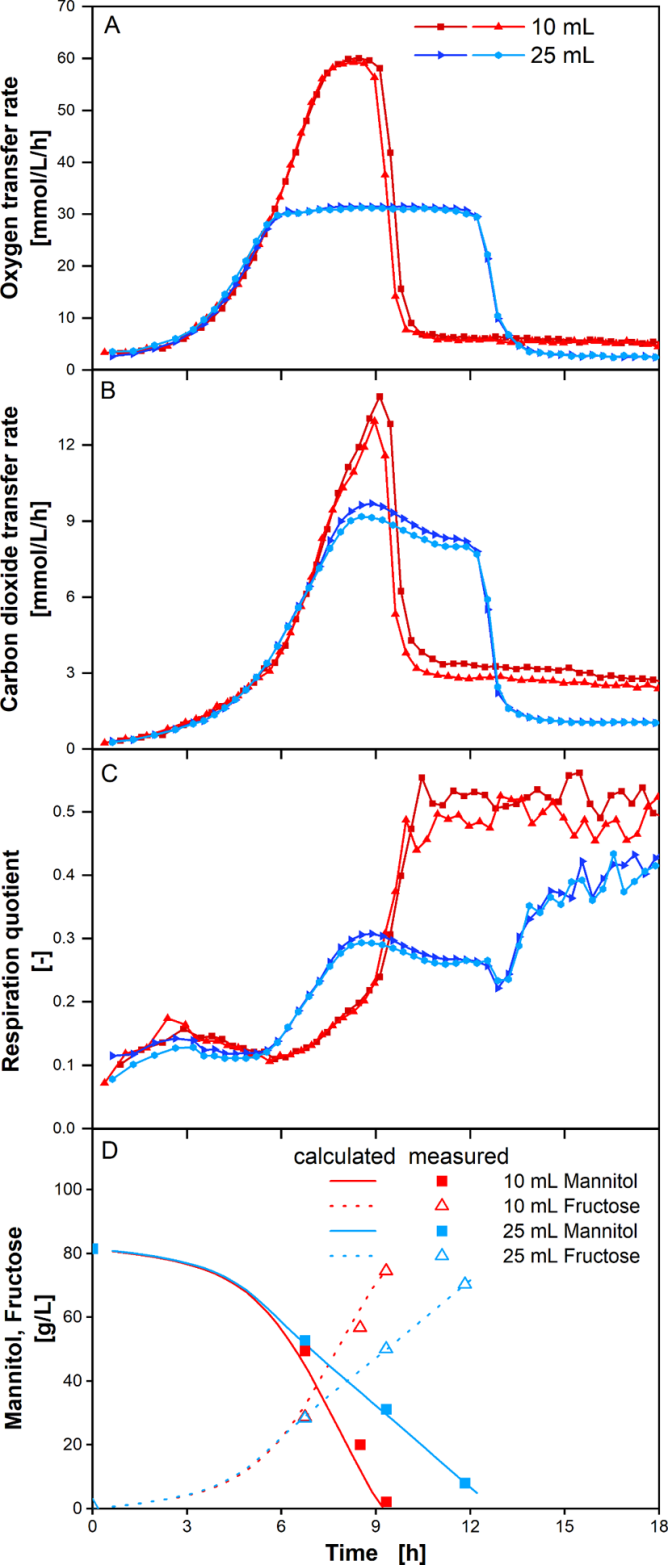
Respiration activity of *G. oxydans* at varying oxygen limitations. OTR, CTR, RQ and sugar concentrations over time of *G. oxydans* 621 H ΔhsdR in Jülich-medium containing 80 g/L mannitol as C-source at two different filling volumes (10 mL and 25 mL). Experiments were performed as duplicate measurements in two individual shake flasks. Time on x-Axis is shifted at some curves for clarity: -0.25 h for 10 mL red square, + 0.25 h for 10 mL red triangle. **A** OTR **B** CTR **C** RQ **D** Measured (square and triangle symbols) and model predicted (solid and dotted lines) sugar concentrations. Model based on total oxygen consumption, total carbon dioxide production and reaction stoichiometry (see Additional file 4); mannitol (measurement: closed squares, model prediction: solid line); fructose (measurement: open triangles, model prediction: dotted line). Sugar measurements are single point measurements. Calculated values are based on average total oxygen consumption and average total carbon dioxide production from two individual shake flasks (see Additional file 3). Operation conditions: *n* = 350 rpm, d_0_ = 50 mm, V_L_ = 10 and 25 mL, 250 mL shake flask, T = 30 °C, t_meas_ = 12 min reduced to t_meas_ = 6 min after 3 h

*G. oxydans* was chosen, as it is known for its capability of incomplete oxidation of various C-sources, due to the abundance of a large variety of dehydrogenases. Little C-source is used for growth, which results in high yields of industrially relevant products, such as natural sweeteners [47]. Here, *G. oxydans* was cultivated in complex medium with 80 g/L mannitol as primary C-source. Similar cultivations (lower mannitol concentration and different shaking conditions) have been conducted by Richhardt et al. [48] for the investigation of *G. oxydans* metabolic pathways on mannitol. The data in the current study emphasizes the potential of online respiration measurements. Figure 5A presents the course of the OTR for two different filling volumes. At 6 h (25 mL filling volume) and 7.5 h (10 mL filling volume) all cultivations enter a period of stationary OTR, which is indicating oxygen limitation [17]. Maximum oxygen transfer is higher at low filling volume, resulting in an overall faster conversion of the C-source and a shorter period of oxygen limitation. Primary C-source depletion is often indicated by a sharp decrease in OTR and CTR [17]. This occurs at 9 h (10 mL filling volume) and 12 h (25 mL filling volume). Then, respiration continues at a lower level. Though these conditions are not oxygen limited anymore, OTR and CTR are significantly higher when filling volume is low. Further investigation of this phenomenon was not part of this study. Richhardt et al. [48] already described two growth phases for *G. oxydans* on mannitol as primary C-source. Growth on mannitol yielded high fructose titers and biomass yield (see Additional File 3). In the subsequent growth phase on fructose, biomass yield was lower, and fructose was converted to keto-fructose, which is in good agreement with the observed reduced respiration level [48]. In addition to their findings, during oxygen limiting conditions (from approx. 6–7.5 h onwards), a change in metabolism is indicated by an increasing RQ value. RQ rises from approx. 0.1 under oxygen unlimited conditions to an RQ-plateau of approx. 0.3 at 25 mL filling volume. As cells keep growing under oxygen limited conditions, but the total oxygen transfer is limited, less oxygen per cell is available as the cultivation proceeds. This might cause a slowly increasing RQ rather than an abrupt jump. When an RQ of approx. 0.3 is reached, growth already slows down, as seen from OD_600_ measurements, and little acetate formation occurs even though mannitol is still present (see Additional file 3). However, at low filling volume of 10 mL, this phase is not entered, probably as mannitol is exhausted before an RQ of 0.3 is reached.

Mannitol is mainly converted to fructose at high yield (Fig. 5D) and peak fructose titers are reached at complete mannitol depletion. When mannitol is fully depleted, respiration does not drop to 0 mmol/L/h, but remains at a certain level, the less filling volume the higher respiration. Growth on fructose and conversion of fructose to 5-ketofructose could be responsible for this respiration, as indicated by a decreasing fructose concentration (Additional file 3) [48]. The RQ value during this phase of the cultivation is higher than for growth on mannitol, indicating that oxidation of fructose to 5-ketofructose is not as dominant as oxidation of mannitol to fructose.

Figure 5D shows the predicted mannitol consumption and fructose production. The concentrations were calculated from total consumed oxygen and total produced carbon dioxide under the assumption that CO_2_ is only produced for biomass growth and maintenance, while roughly the same amount of oxygen is consumed (assumption RQ = 0.92 for biomass growth and maintenance, see Additional file 4 Eq. 9). Elemental composition of *G. oxydans* for stoichiometric calculations was presented by Herweg et al. [47]. Additional consumed oxygen is used to oxidize mannitol to fructose (Additional file 4, Eq. 10). The model-predicted values for mannitol and fructose very well match the HPLC derived values. This highlights the ability of off-gas analysis for stoichiometric analysis of cell metabolism.

### *E. coli* tuner protein expression

**Fig. 6:**
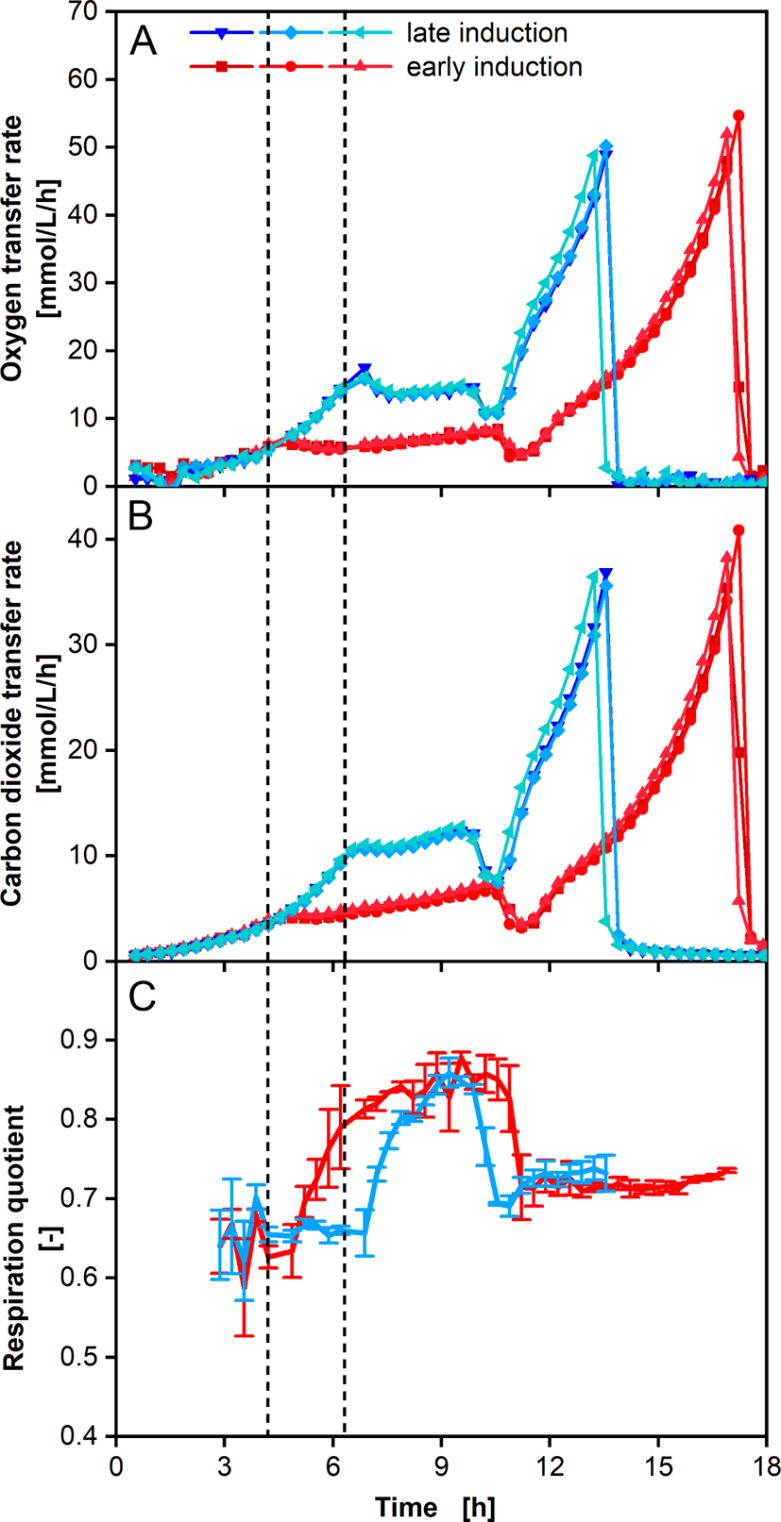
Respiration activity measurements for arabinose induced *E. coli* Tuner cultivations.**A** OTR, **B** CTR and **C** RQ over time of *E. coli* Tuner in 0.2 M Wilms-MOPS medium containing 7.5 g/L glycerol. *E. coli* was induced with 10 mM final arabinose concentration after 4.5 h, when an OTR of 5 mmol/L/h was reached (first dashed line), (red curves) and after 6.5 h, when an OTR of 14 mmol/L/h was reached (second dashed line) (blue curves). Experiments were performed as triplicate measurements in three individual shake flasks. Operation conditions: *n* = 350 rpm, d_0_ = 50 mm, V_L_ = 10 mL, 250 mL shake flask, T = 37 °C, t_meas_= 6 min. RQ is displayed for OTR values larger than 3 mmol/L/h

An *E. coli* Tuner strain was grown on Wilms MOPS medium containing 7.5 g/L glycerol, as described by Ihling et al. [49]. Results are presented in Fig. 6. When an OTR of 5 mmol/L/h was reached, the first three cultivations were induced with arabinose, while the next three cultivations were induced after they reached an OTR of 14 mmol/L/h. Increase of both, OTR and CTR, is slowed down after induction with arabinose. This is caused by the phenomenon of metabolic burden [50–52]. During the induction phase, arabinose is metabolized, and recombinant protein is produced. When arabinose is used up, the metabolism switches back to the remaining glycerol left in the medium. This moment is indicated by a distinctive downward kink in OTR and CTR after 10 h (late induction) and 11 h (early induction), respectively. Unlimited exponential growth is observed from OTR and CTR data in this last phase, before glycerol depletion occurs at 13 h (late induction) and 17 h (early induction), respectively. The measured OTR and CTR data reveals high reproducibility between individual flasks and high data resolution at each individual flask. Only minor temporal shifts, most likely resulting from the manual preparation of the cultivation, are visible. High resolution, even at low respiration, also results in good resolution in RQ data. The metabolic switch from growth on glycerol (RQ at approx. 0.67, see Additional file 4 Eq. 12) to conversion of arabinose is clearly observable. The RQ remains at a low level for approx. 20 min after induction, indicating that this time is needed to switch metabolism to recombinant protein expression and arabinose consumption. Given that prior to protein translation intracellular processes like mRNA transcription occur, this assumption appears reasonable. About 20 min after induction, the RQ increases steadily to approx. 0.85 within several hours. For growth or protein expression purely on arabinose, an RQ of approx. 0.94 would be expected (see Additional file 4 Eq. 13). The elemental composition of *E. coli* used for the calculation is described by von Stockar et al. [53]. Thus, an RQ of 0.85 indicates simultaneous consumption of both arabinose and glycerol. Both, the delay in arabinose uptake and simultaneous consumption of both C-sources is in perfect accordance with HPLC data presented by Ihling et al. [49]. The shown data illustrates reproducibility and resolution of the evaluated measurement device. Different metabolic phases are clearly distinguishable and the combination of OTR and CTR measurement and calculation of the RQ from these measurements revealed, which carbon sources were metabolized at which time. This can help finding optimal induction strategies, but also eases finding optimal sampling points to determine maximum levels of recombinant protein expression. This way, offline sampling and the manual work that is typically associated with it can be minimized. Ihling et al. [49] previously performed the same experiment in a RAMOS. OTR, CTR and RQ data is presented in Additional file 5 for comparison to the data obtained with TOM in Fig. 6. OTR data shows similar signal to noise behavior. CTR and resulting RQ show higher noise and RQ does not reflect the metabolic changes that could be differentiated with the TOM.

### Comparison to state of the art technology

Table 1 is specifying main differences and similarities between state of the art measurement systems for off-gas analysis in shake flasks. The TOM measurement principle is based on the RAMOS technology, using recurrent measurement phases of stopped aeration, to determine OTR and CTR from the change in oxygen and CO_2_ partial pressure [17]. By adapting the duration of the measurement phase, the precision of the measurement can be adapted to the respiration activity of the culture. This is only possible with an actively aerated shake flask. The technologies presented by Ge et al., Takahashi et al. (CDMSS) and also the BCpreFerm are only measuring oxygen and CO_2_ partial pressure in the gas phase instead, presenting a major difference to the TOM system [15, 16, 55]. CDMSS and BCpreFerm are using passively ventilated shake flasks. However, the BCpreFerm also converts partial pressures to OTR and CTR, based on the estimated ventilation through a membrane closure on top of the shake flask [55]. The novelty of the TOM approach is the combination of the RAMOS principle with the sensors placed in a bypass, as suggested by Takahashi et al. [15]. In this way, robust sensor operation is secured (as the sensors are non-shaken) and the Δ-pressure sensor used by the RAMOS technology for indirect CTR measurement could be replaced by an NDIR sensor, showing superior performance, as presented in Fig. 2. Additional sensors could potentially be included, showing another advantage of the bypass type system [15]. Using a bypass system, different types and sizes of standard shake flasks can be connected. In contrast, RAMOS and the BCpreFerm system require special shake flasks to hold the sensors [54, 55]. For the system presented by Ge et al., sensors need to be placed inside the shake flask prior to usage [16]. From the operation perspective, robustness of the measurement system was strongly increased, as (A) flasks monitored with the RAMOS technology have to be air tight for accurate CTR measurement, which is difficult to achieve and maintain, (B) blockage or dysfunction of the outlet valve (see additional file 2 A) is banned, as it is replaced by an open vent hole and (C) oxygen sensor failure due to shaking conditions can be avoided. These points have in the past been evaluated as major drawbacks during operation of the RAMOS system.

**Table 1:** Comparison of different technologies for off-gas analysis in shake flasks

Measurement System	Aeration	Data Output	Measurement principle	Sensor technology	Sensor placement
RAMOS [55]	Active	OTR/CTR	Partial pressure change in measurement phase	Electrochemical/Pressure	Flask
CDMSS [15]	Passive	O_2_/CO_2_ in the gas phase	Partial pressure	Fluorescence/NDIR	Bypass
Triple disposable non-invasive sensors [16]	Passive or active	O_2_/CO_2_ in the gas phase	Partial pressure	Fluorescence	Flask or Off-gas stream
BCpreFerm [54]	Passive	O_2_/CO_2_ in the gas phase	Partial pressure	Zirconium dioxide/NDIR	Flask
TOM	Active	OTR/CTR	Partial pressure change in measurement phase	Electrochemical/NDIR	Bypass

## Discussion

This study introduced a novel monitoring system for OTR and CTR in shake flasks. Measurement is based on electrochemical oxygen sensors and NDIR carbon dioxide sensors. In this study, 250 mL Erlenmeyer flasks were used, as they represent a widely used type of shake flask. However, other shake flask sizes and geometries (even baffled) flasks could also be used, when an appropriate lid is available including inlet port, outlet port and vent hole. Care must be taken that the gas in the flask headspace is always well mixed to result in a homogenous gas composition. This way, the sensors can follow the dynamic changes of the O_2_ and CO_2_ partial pressures during the measurement phases. Operating conditions, such as large flasks, very low filling volumes, no- or very low shaking or solid (non-shakable) substrates, could potentially decrease measurement performance, as these operating conditions could result in a non-homogenous gas phase. These operating conditions were, therefore, not covered during this study.

Aeration was performed with a self-priming microfluidic pump, which showed reliable performance over a period of at least three years (+-10% of set flow, field experience, data not shown), which is seen not critical to measurement performance.

The gas outlet (open vent hole) was designed as a small diameter tube (1.6 mm diameter, 50 mm long) to function as a diffusion barrier for ambient air. The setup must not be gas tight for accurate measurement and an outlet valve is dispensable. However, it cannot be excluded that traces of ambient air could enter the shake flask. At RQ < 1 ambient air is sucked in.

In this study, the differential-pressure sensors that were previously used for the RAMOS, were replaced by NDIR CO_2_ sensors. These sensors were demonstrated to have superior resolution compared to Δp sensors. However, NDIR CO_2_ sensor sensitivity decreases with higher CO_2_ partial pressure. Thus, CO_2_ partial pressure above 16,000 Pa (approx. 16% v/v) should not be exceeded for the CO_2_ sensor applied in this study for good resolution. Processes with low aeration rates (< 0.15 vvm) in combination with high filling volumes (> 20% relative filling volume) and strong microbial CO_2_ production could potentially exceed this partial pressure.

Homogeneously mixed yeast and bacterial cultures were used in non-baffled flasks to demonstrate the application potential of the TOM system. Furthermore, off-gas analysis has immense potential for cultivations that can hardly be followed online due to non-homogeneity or opacity of the medium, such as highly viscous, foaming, splashing medium, solid substrates or adherently growing cells. Mammalian cell culture cultivations, which are typically characterized by low respiration activities (< 2 mmol/L/h), represent another interesting application for the presented TOM system, as measurement resolution can be increased by long measurement times [18–20, 46, 56].

## Conclusions

The TOM is an off-gas analysis device for shake flasks, measuring OTR, CTR, RQ, total consumed oxygen and total produced carbon dioxide. Sensor performance and measurement setup were tested against state of the art RAMOS. A robust setup for evaluation of accuracy and resolution of the TOM was presented. A combination of high resolution O_2_ and CO_2_ sensors in combination with a dynamic measurement method led to high reproducibility among individual shake flasks and highest CTR resolution reported so far for shake flask applications. Combination of both measures (OTR and CTR) enabled quantitative analysis of consumed substrates and formed products. Also, metabolic switches from growth to protein production were easy to determine. As the TOM electronics and also the sensors are non-shaken, high robustness is combined with flexibility, as a variety of different shake flask types and sizes could be connected with an appropriate lid (different types and sizes).

## Methods

### Developed measurement system

A schematic overview of the developed measurement system is shown in Fig. 1A. The shake flask is aerated via a self-priming micropump (mp6, Bartels, Germany) with air from the incubator chamber. This air passes an inlet valve before entering the shake flask. Off-gas leaves the shake flask via a vent hole (green path in Fig. 1A). A recycle pump (mp6, Bartels, Germany) continuously withdraws gas from the flask headspace and passes this gas through an a non-dispersive infrared (NDIR) CO_2_ sensor (Alphasense Ltd., UK) and electrochemical oxygen sensor (Maxtec, US), before recycling the gas back to the flask headspace (blue path in Fig. 1A and B). Recycling does not alter the molar balance of oxygen and carbon dioxide in the shake flask headspace. The flow of the recycle pump was set to approx. 15 mL/min. Homogenous mixing of the headspace is essential which is solely achieved by the shaking movement, not by the air flow of the recycle pump. Experiments using smoke as a tracer gas previously showed that the headspace of the standard shake flask can be regarded as well mixed under the shaking conditions used in this study (d_0_ = 50 mm, *n* > 200 rpm) [57]. Takahashi et al. previously reported about CO_2_ gradients in Sakaguchi flasks at low shaking or static conditions [58]. To avoid this problem, in the TOM, dead zones (e.g. in the shake flask neck) are avoided by the shake flask lid design, which is completely filling the shake flask neck. Also, static or slow shaking conditions should be avoided.

OTR and CTR is measured in recurrent measurement phases (see Fig. 1C), as presented before for the RAMOS [17, 54]. In a measurement phase, the inlet valve (3/2 way solenoid valve, Bürkert, Germany) is closed to stop aeration and - consequently - due to the respiration of organisms or cells, the oxygen partial pressure decreases and the carbon dioxide partial pressure increases. The change in partial pressure is then converted to OTR and CTR, respectively (see Eqs. 3 and 4). After the measurement phase (typically t_meas_ = 6 min), a phase of strong aeration (flushing, typically 1 min) accounts for the depletion of oxygen and rise in carbon dioxide from the headspace during the measurement phase, before re-entering the aeration phase (typically 13 min). In the aeration phase, the aeration rate can be varied to mimic the aeration in passively ventilated shake flasks with different flask stoppers. In this way, an equal headspace gas composition between passively ventilated and actively aerated flasks is achieved [25, 57]. This is crucial for the comparability of results from monitored and conventional flasks. In this study, the aeration rate was set to 11 mL/min. This makes the gas composition of the headspace of the actively aerated shake flasks (TOM) comparable to those in standard shake flasks, ventilated via cotton plug as sterile closure, as presented by Mrotzek et al. [25]. Electronics, sensors and pumps are placed in a housing above the shaker tray (sensor module). This design reduces the risk of damage through intense shaking. Flexible tubing (Tygon, 1.6 mm ID) connects the sensor module to the shake flasks (Fig. 1D). The gas volume of the total bypass is approx. 1.5 mL resulting in fast response at 15 mL/min flow.

### Online monitoring

Pre-cultures and main-cultures of cultivations of model organisms were monitored for OTR and CTR with an in-house developed TOM (Fig. 1), if not stated differently. For performance measurement of sensors during development, a modified in-house developed TOM was used, comprising a switchable solenoid valve instead of an open vent hole. Usage of those tools is stated whenever applied. All cultivations were performed in an incubator shaker from Kuhner AG, Switzerland (model ISF1-X).

### *Saccharomyces cerevisiae* cultivations

*S. cerevisiae* DSM70449 was cultivated in YEP-medium containing 10 g/L yeast extract, 10 g/L peptone and 20 g/L glucose. The preculture was cultivated in 250 mL shake flasks containing V_L_ = 50 mL culture broth at *n* = 200 rpm, d_0_ = 5 cm at T = 30 °C. The pre-culture was stopped in mid-exponential growth at OD_600_ between 3 and 4.5. The main culture was cultivated in 250 mL shake flasks containing V_L_ = 25 mL culture broth at *n* = 200 rpm, d_0_ = 5 cm at T = 30 °C, start OD_600_ = 0.2.

### *Gluconobacter oxydans* cultivations

*G. oxydans* 621 H ΔhsdR was cultivated in a complex mannitol medium (Jülich Medium) containing 5 g/L yeast extract, 1 g/L KH_2_PO_4_, 1 g/L (NH_4_)_2_SO_4_, 2.5 g/L MgSO_4_·7H_2_O; 80 g/L mannitol, 0.05 g/L cefoxin [59]. pH was adjusted to 6 with hydrochloric acid. The pre-culture was cultivated in 250 mL shake flasks containing V_L_ = 20 mL culture broth at *n* = 350 rpm, d_0_ = 5 cm at T = 30 °C. The pre-culture was stopped in mid-exponential phase at OD_600_ between 0.7 and 1.7. The pre-culture broth was centrifuged, the supernatant was discarded, and cells were resuspended in fresh medium. The main culture was cultivated in 250 mL shake flasks at varying filling volumes of V_L_ = 10 mL and V_L_ = 25 mL culture broth at *n* = 350 rpm, d_0_ = 5 cm at T = 30 °C, initial OD_600_ = 0.1. Offline samples were taken from normal flasks that run in parallel under the same conditions.

### *Escherichia coli* cultivations

An *E. coli* Tuner strain was purchased from Novagen and transformed with an arabinose-inducible plasmid [49]. The first pre-culture was cultivated in complex 2xYT-medium containing 16 g/L tryptone (Carl Roth GmbH, Germany), 10 g/L yeast extract (Carl Roth GmbH, Germany), 5 g/L NaCl, and 10 mM calcium (supplemented as CaCl_2_ ∙ 2 H_2_O). The pH was adjusted to 7.0 ± 0.2 using 5 M sodium hydroxide solution. Afterwards, the medium was sterilized at 121 °C for 20 min. Main culture and second pre-culture was cultivated in modified Wilms-MOPS mineral medium according to Wilms et al. [60]. Medium preparation and cultivation procedure was conducted as described by Ihling et al. [49]. The medium consisted of 7.5 g/L glycerol, 6.98 g/L (NH_4_)_2_SO_4_, 3 g/L K_2_HPO_4_, 2 g/L Na_2_SO_4_, 41.85 g/L (= 0.2 M) (N-Morpholino)-propanesulfonic acid (MOPS), 0.5 g/L MgSO_4_∙7H_2_O, 0.01 g/L thiamine hydrochloride, 0.1 g/L ampicillin (resistance on product plasmid), 0.03 g/L kanamycin (resistance on transporter plasmid), 1 ml/L trace element solution [0.54 g/L ZnSO_4_∙7H_2_O, 0.48 g/L CuSO_4_∙5H_2_O, 0.3 g/L MnSO_4_∙H_2_O, 0.54 g/L CoCl_2_∙6H_2_O, 41.76 g/L FeCl_3_∙6H_2_O, 1.98 g/L CaCl_2_ ∙ 2H_2_O, 33.4 g/L Na_2_EDTA (Titriplex III). The pH-value was adjusted to 7.3 using 5 M NaOH. All components were mixed from sterile stock solutions that were separately sterilized by autoclaving or sterile filtration. The medium was prepared directly before use. The concentration of calcium (supplemented as CaCl_2_∙2H_2_O) and citrate (supplemented as sodium citrate) was 10 mM each. Both were added as the last components from sterile filtered stock solutions. Induction of the transporter plasmid was performed by the addition of 1 mM isopropyl-β-D-thiogalactoside (IPTG; 1 M stock solution) at the beginning of the cultivation and the product plasmid was induced by 10 mM L-(+)-arabinose (2.5 M stock solution) during the cultivation.

Main and pre-cultures were cultivated in 250 mL shake flasks containing V_L_ = 10 mL medium at *n* = 350 rpm, d_0_ = 5 cm. The first pre-culture in complex 2xYT-medium was incubated at T = 37 °C for 3–4 h starting with an OD_600_ of 0.035. The second pre-culture was incubated at T = 30 °C in mineral Wilms-MOPS medium with an initial OD_600_ of 0.01 and cultivated for 16 h. The main culture was incubated at T = 30 °C in mineral Wilms-MOPS medium with a starting OD_600_ of 0.1.

### Performance evaluation

Performance measurements were conducted as described by Schulte et al. [9]. OTR and CTR were measured while O_2_ depleted and CO_2_ enriched gas (Gas 1: 79% N_2_, 17% O_2_, 4% CO_2_ or Gas 2: 79% N_2_, 5% O_2_, 16% CO_2_) was continuously fed to the shake flask with a mass flow controller. This gas flow imitates respiration as O_2_ level decreases and CO_2_ level increases in the shake flask headspace. Different set mass flow controller flow rates imitate different levels of OTR and CTR (0, 1.56, 3.13, 6.25 nmL/min Gas 1 and 2, 4 nmL/min/flask Gas 2). These set respiration rates are compared to measured values. 250 mL shake flasks were shaken at *n* = 180 rpm, d_0_ = 5 cm, V_L_ = 10 mL water at T = 30 °C and 11 mL/min aeration with air. Set OTR and CTR ranged from 0 mmol/L/h to 57 mmol/L/h (see Table 2). Eight individual flasks were monitored and 4 measurements were taken per flask.

**Table 2:** Gas composition and flow setpoints for performance measurements

Gas composition [%O_2_/%CO_2_]	17 / 4	17 / 4	17 / 4	17 / 4	5 / 16	5 / 16
Gas feed flow rate *F*_Feed_ [nmL/min]	0	1.56	3.12	6.25	2	4
Resulting respiration rate OTR_set_, CTR_set_ [mmol/L/h]	0	9.94	11.91	18.29	34.32	56.93

The course of the oxygen and CO_2_ partial pressure in the shake flask was simulated according to Eqs. 14 and 15 for each feed flow rate. According respiration rates were then calculated with Eqs. 3 and 4.

14 $$\:\frac{dp}{dt}=\frac{{F}_{in}}{{V}_{g}}\times\:{p}_{Feed}\:-\frac{{F}_{out}^{*}}{{V}}_{{g}}\times\:{p}$$

15 $$\:{{p}}_{{s}{t}{a}{r}{t}}=({{F}}_{{F}{e}{e}{d}}\times\:{{p}}_{{F}{e}{e}{d}}+{{F}}_{{i}{n}}\times\:{{p}}_{{i}{n}})/{{F}}_{{o}{u}{t}}$$

## Electronic supplementary material

Below is the link to the electronic supplementary material.

Supplementary Material 1: Development of TOM from RAMOS. The timeline illustrates which system has been used for the according experiment. TOM is based on the RAMOS measurement principle. First different CO_2_ sensor technologies have been compared using a hybrid system (see experiment in Fig. 2). Next, the gas outlet configuration was compared (see experiment in Fig. 3). The closed hybrid system using an outlet valve was compared to the final TOM system with open vent hole. All further experiments were conducted with the final TOM system

Supplementary Material 2: Flow scheme of RAMOS and modified TOM for comparison of NDIR CO_2_ sensor and Δp sensor for CTR measurement **A** Simplified flow scheme of RAMOS with Δp sensor, oxygen sensor, inlet and outlet valve. Detailed information is available from Anderlei et al. [17, 54] **B** Simplified flow scheme of a TOM device that was modified by including an additional Δp sensor and outlet valve (purple). This setup was used to compare both technologies (RAMOS and TOM). During the measurement phase, inlet valve and outlet valve are closed. At RQ > 1 (CTR > OTR) pressure in the shake flask will increase. At RQ < 1 (CTR < OTR) pressure will decrease. The method is described in detail in Anderlei et al. [17].The CO_2_ sensor detects changes in CO_2_ partial pressure that are directly converted to CTR readings

Supplementary Material 3: Total consumed oxygen, total produced carbon dioxide (online measured) and mannitol, fructose, acetate, pH and OD_600_ (offline sampling) over time of *G. oxydans* 621 H ΔhsdR. Cultivation in Jülich-medium containing 80 g/L mannitol as C-source at two different filling volumes (10 mL and 25 mL). This data refers to the experiment shown in Fig. 5. **A** Total consumed oxygen and total produced carbon dioxide. This data is derived from OTR and CTR data presented in Fig. 5 A and B. Measurements were performed in two individual shake flasks. Time on x-Axis is shifted at some curves for clarity: -0.25 h for 10 mL red square, + 0.25 h for 10 mL red triangle. **B** Mannitol (solid line), fructose (dotted line), acetate (dashed line). **C** pH (dotted line), OD_600_ (solid line). Offline sampled data was determined from flasks that were run in parallel to online monitored shake flasks. All data points are single measurements

Supplementary Material 4: Stoichiometric equations and resulting expected RQ values for *E. coli* and *G. oxydans*

Supplementary Material 5: Respiration activity measurements for arabinose induced *E. coli* Tuner cultivations with RAMOS. OTR data of this dataset was presented before by Ihling et al. [49] **A** OTR, **B** CTR and **C** RQ over time of *E. coli* Tuner in 0.2 M Wilms-MOPS medium containing 7.5 g/L glycerol. *E. coli* was induced with 10 mM final arabinose concentration after 3 h, when an OTR of 5 mmol/L/h was reached (first dashed line), (red curves) and after 5 h, when an OTR of 17 mmol/L/h was reached (second dashed line) (blue curves). Experiments were performed as duplicate measurements in two individual shake flasks. Operation conditions: *n* = 350 rpm, d_0_ = 50 mm, V_L_ = 10 mL, 250 mL shake flask, T = 37 °C, t_meas_ = 6 min. RQ is displayed for OTR values larger than 3 mmol/L/h

## Data Availability

No datasets were generated or analysed during the current study.

## Abbreviations

CTR: Carbon dioxide transfer rate [mmol/L/h
DOT: Dissolved Oxygen Tension [%]
d_0_: Shaking diameter [cm]
F_Feed_: Gas feed flow rate [m^3^/h]
F_in_: Incoming gas flow in aeration phase [m^3^/h]
F_out_: Outgoing gas flow in aeration phase [m^3^/h]
F_out_*: Outgoing gas flow in measurement phase [m^3^/h]
*E. coli*: Escherichia coli Tuner
*G. oxydans*: Gluconobacter oxydans 621 H ΔhsdR
k_L_a: Liquid side oxygen mass transfer coefficient [1/h]
L_O2_: Oxygen solubility [mmol/L/Pa]
n: Shaking frequency [1/min]
NDIR: Non-dispersive infrared
OTR: Oxygen transfer rate [mmol/L/h
p_CO2_: CO_2_ partial pressure [Pa]
p_Feed_: Partial pressure in feed [Pa]
p_O2_: O_2_ partial pressure [Pa]
p_O2,cal_: O_2_ partial pressure during calibration [Pa]
RQ: Respiration quotient
*S. cerevisiae*: Saccharomyces cerevisiae DSM70449
R: Gas constant [J/mol/K]
T: Temperature [K]
V_L_: Filling volume [m^3^]
V_G_: Gas volume [m^3^]
