# Supplementary figures and images for: A new approach to off-gas analysis for shaken bioreactors showing high CTR and RQ accuracy
Source: J Biol Eng. 2025 Jan 28;19:11. doi: 10.1186/s13036-025-00480-5 (PMC11776160; doi:10.1186/s13036-025-00480-5)

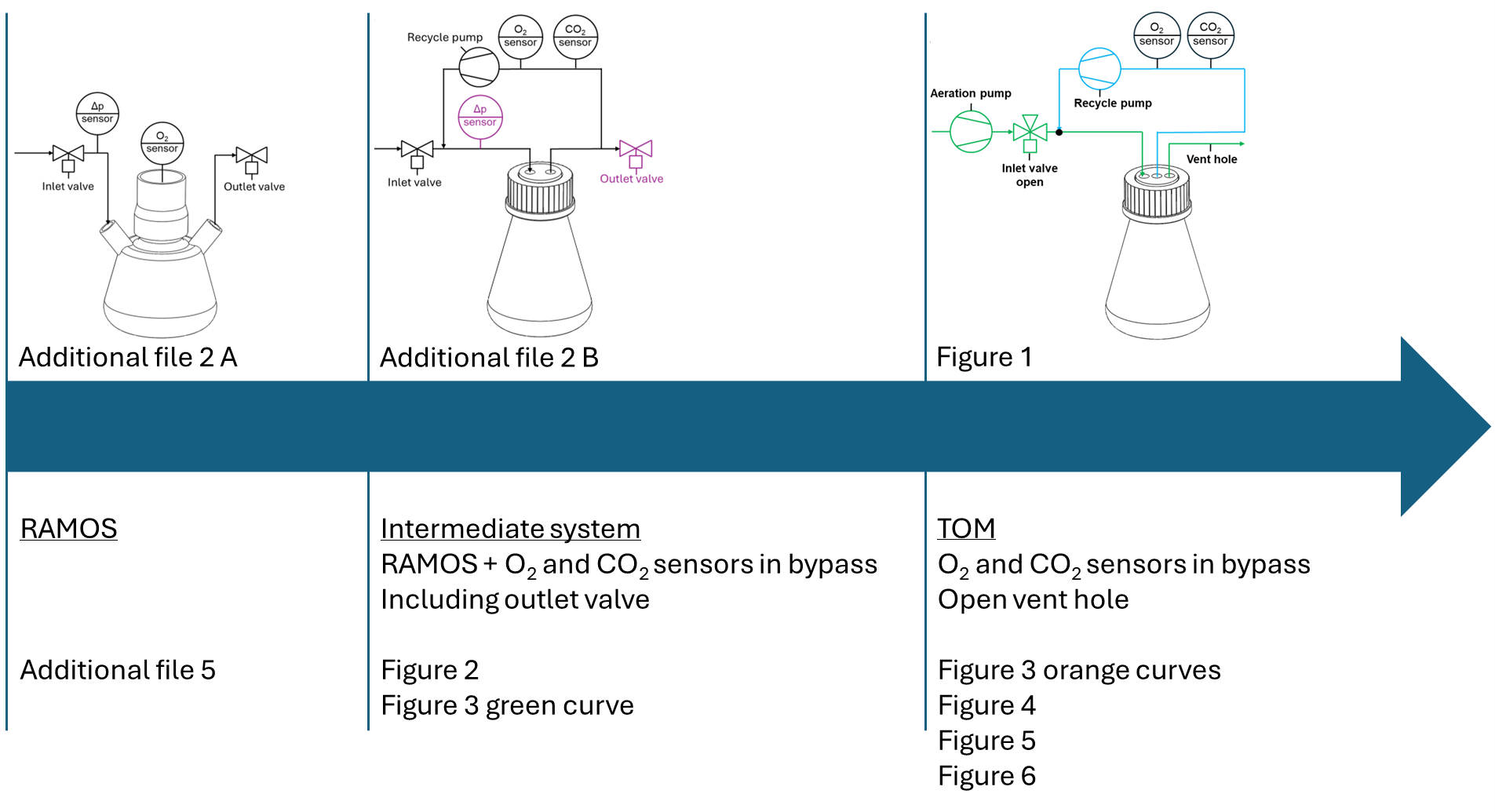

Supplement: Supplementary file 1 — Supplementary Material 1: Development of TOM from RAMOS. The timeline illustrates which system has been used for the according experiment. TOM is based on the RAMOS measurement principle. First different CO2 sensor technologies have been compared using a hybrid system (see experiment in Fig. 2). Next, the gas outlet configuration was compared (see experiment in Fig. 3). The closed hybrid system using an outlet valve was compared to the final TOM system with open vent hole. All further experiments were conducted with the final TOM system [file 13036_2025_480_MOESM1_ESM.tif]

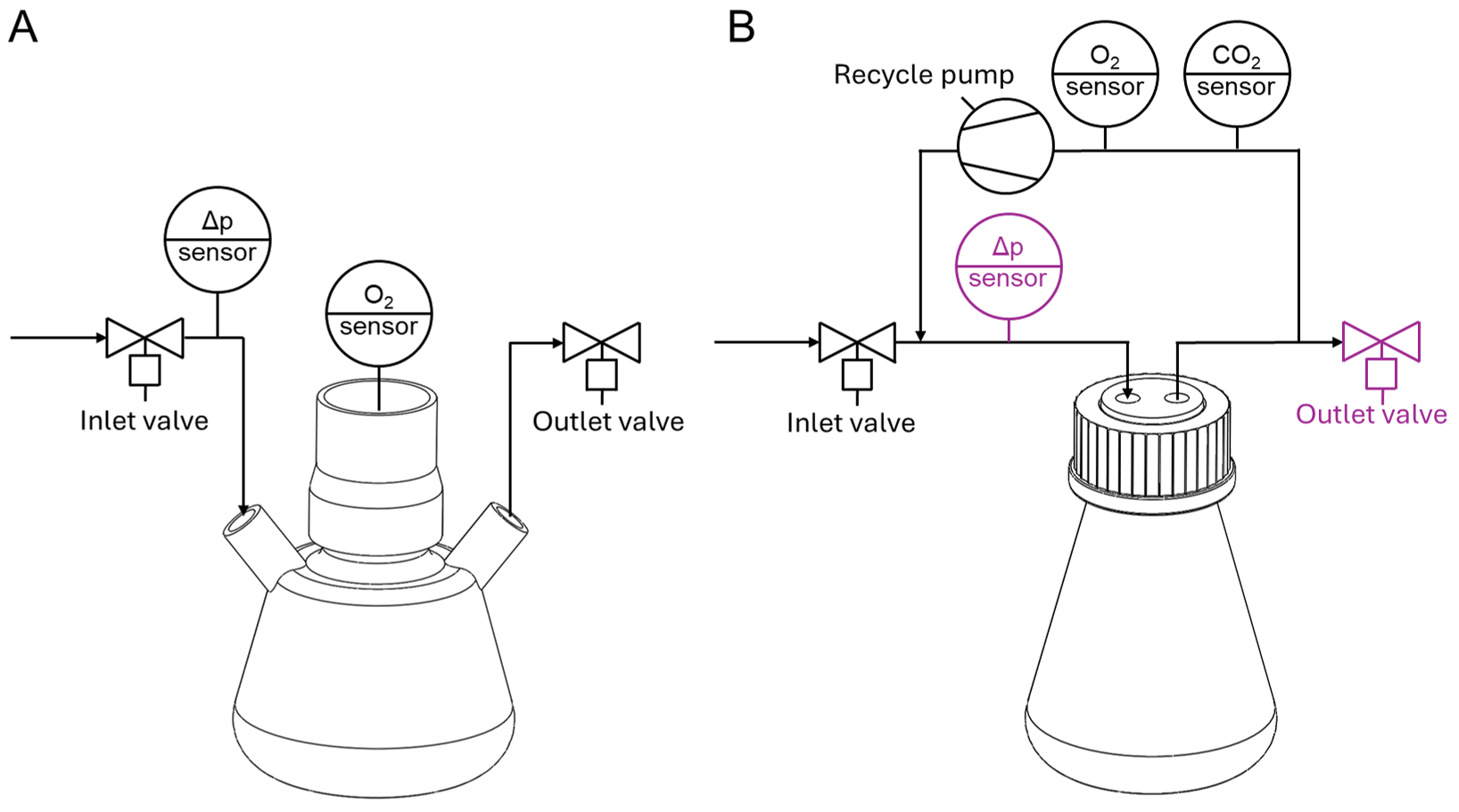

Supplement: Supplementary file 2 — Supplementary Material 2: Flow scheme of RAMOS and modified TOM for comparison of NDIR CO2 sensor and Δp sensor for CTR measurement A Simplified flow scheme of RAMOS with Δp sensor, oxygen sensor, inlet and outlet valve. Detailed information is available from Anderlei et al. [17, 54] B Simplified flow scheme of a TOM device that was modified by including an additional Δp sensor and outlet valve (purple). This setup was used to compare both technologies (RAMOS and TOM). During the measurement phase, inlet valve and outlet valve are closed. At RQ > 1 (CTR > OTR) pressure in the shake flask will increase. At RQ < 1 (CTR < OTR) pressure will decrease. The method is described in detail in Anderlei et al. [17].The CO2 sensor detects changes in CO2 partial pressure that are directly converted to CTR readings [file 13036_2025_480_MOESM2_ESM.tif]
