# Supplementary material for: A new approach to off-gas analysis for shaken bioreactors showing high CTR and RQ accuracy
Source: J Biol Eng. 2025 Jan 28;19:11. doi: 10.1186/s13036-025-00480-5 (PMC11776160; doi:10.1186/s13036-025-00480-5)
Supplement: Supplementary file 4 — Supplementary Material 4: Stoichiometric equations and resulting expected RQ values for E. coli and G. oxydans [file 13036_2025_480_MOESM4_ESM.pdf]

Additional file 3 Stoichiometric equations and resulting expected RQ values for *E. coli* and *G. oxydans.*

G. oxydans growth on mannitol ($C_6H_{14}O_6$) $Y_{x/s}$ = 0.21 (Elemental composition of *G. oxydans* from [47])

$$1\ C_6H_{14}O_6 + 4.72\ O_2 + 0.34\ NH_4 = \ 1.09\ CH_{1.78}O_{0.38}N_{0.2} + 6.17\ H_2O + 4.31\ CO_2 \quad (9)$$

$$RQ = 0.92$$

G. oxydans conversion of mannitol ($C_6H_{14}O_6$) to fructose ($C_6H_{12}O_6$)

$$1\ C_6H_{14}O_6 + 0.5\ O_2 = 1\ C_6H_{12}O_6 + 1\ H_2O \quad (10)$$

$$RQ = 0$$

G. oxydans conversion of fructose ($C_6H_{12}O_6$) to 5-keto-fructose ($C_6H_{10}O_6$)

$$1\ C_6H_{12}O_6 + 0.5\ O_2 = 1\ C_6H_{10}O_6 + 1\ H_2O \quad (11)$$

$$RQ = 0$$

E. coli growth on glycerol ($C_3H_8O_3$) $Y_{x/s}$ = 0.5 (Elemental composition of *E. coli* from [53])

$$1\ C_3H_8O_3 + 1.73\ O_2 + 0.44\ NH_4 = \ 1.84\ CH_{1.77}O_{0.49}N_{0.24} + 3.25\ H_2O + 1.16\ CO_2 \quad (12)$$

$$RQ = \frac{CO_2}{O_2} = 0.67$$

E. coli growth on arabinose ($C_6H_{12}O_6$) $Y_{x/s}$ = 0.5 (Elemental composition of *E. coli* from [53])

$$1\ C_6H_{12}O_6 + 2.55\ O_2 + 0.87\ NH_4 = \ 3.6\ CH_{1.77}O_{0.49}N_{0.24} + 4.54\ H_2O + 2.4\ CO_2 \quad (13)$$

$$RQ = 0.94$$
